# Baclofen into the lateral parabrachial nucleus induces hypertonic sodium chloride intake during cell dehydration

**DOI:** 10.1186/1744-9081-9-17

**Published:** 2013-05-03

**Authors:** Everton H Kimura, Lisandra B De Oliveira, José V Menani, João C Callera

**Affiliations:** 1 Department of Basic Sciences, School of Dentistry, UNESP – Univ. Estadual Paulista, Rodovia Marechal Rondom, km 527, Araçatuba, São Paulo, 16018-805, Brazil; 2 Department of Biological Sciences, DECBI-NUPEB, Federal University of Ouro Preto, UFOP, Ouro Preto, Minas Gerais, Brazil; 3 Department of Physiology and Pathology, School of Dentistry, UNESP, Araraquara, São Paulo, Brazil

**Keywords:** Baclofen, Dehydration, Sodium appetite, Thirst, Lateral parabrachial nucleus

## Abstract

**Background:**

Activation of GABA_B_ receptors with baclofen into the lateral parabrachial nucleus (LPBN) induces ingestion of water and 0.3 M NaCl in fluid replete rats. However, up to now, no study has investigated the effects of baclofen injected alone or combined with GABA_B_ receptor antagonist into the LPBN on water and 0.3 M NaCl intake in rats with increased plasma osmolarity (rats treated with an intragastric load of 2 M NaCl). Male Wistar rats with stainless steel cannulas implanted bilaterally into the LPBN were used.

**Results:**

In fluid replete rats, baclofen (0.5 nmol/0.2 μl), bilaterally injected into the LPBN, induced ingestion of 0.3 M NaCl (14.3 ± 4.1 vs. saline: 0.2 ± 0.2 ml/210 min) and water (7.1 ± 2.9 vs. saline: 0.6 ± 0.5 ml/210 min). In cell-dehydrated rats, bilateral injections of baclofen (0.5 and 1.0 nmol/0.2 μl) into the LPBN induced an increase of 0.3 M NaCl intake (15.6 ± 5.7 and 21.5 ± 3.5 ml/210 min, respectively, vs. saline: 1.7 ± 0.8 ml/210 min) and an early inhibition of water intake (3.5 ± 1.4 and 6.7 ± 2.1 ml/150 min, respectively, vs. saline: 9.2 ± 1.4 ml/150 min). The pretreatment of the LPBN with 2-hydroxysaclofen (GABA_B_ antagonist, 5 nmol/0.2 μl) potentiated the effect of baclofen on 0.3 M NaCl intake in the first 90 min of test and did not modify the inhibition of water intake induced by baclofen in cell-dehydrated rats. Baclofen injected into the LPBN did not affect blood pressure and heart rate.

**Conclusions:**

Thus, injection of baclofen into the LPBN in cell-dehydrated rats induced ingestion of 0.3 M NaCl and inhibition of water intake, suggesting that even in a hyperosmotic situation, the blockade of LPBN inhibitory mechanisms with baclofen is enough to drive rats to drink hypertonic NaCl, an effect independent of changes in blood pressure.

## Background

The lateral parabrachial nucleus (LPBN), is a pontine structure located dorsal to the superior cerebellar peduncle (SCP). The LPBN is reciprocally connected to different areas, such as area postrema (AP) and medial portion of the nucleus of the solitary tract (mNTS) in the hindbrain and forebrain areas, such as the paraventricular nucleus of the hypothalamus, central nucleus of amygdala and median preoptic nucleus [1-7]. Cells in the LPBN are activated after ingestion of NaCl solutions by dehydrated rats or by rats that received intragastric load of hypertonic NaCl, suggesting that LPBN cells are activated by visceral or taste signals [8-10]. Therefore, the LPBN may convey signals that ascend from AP/mNTS to forebrain areas involved in the control of fluid and electrolyte balance.

Studies about the involvement of the LPBN in the control of fluid-electrolyte balance have investigated mainly the inhibitory mechanisms for water and NaCl intake and different neurotransmitters or receptors in the LPBN have been shown to be involved with these mechanisms. For example, bilateral injection of the serotonergic antagonist methysergide into the LPBN increased water and 0.3 M NaCl intake induced by different treatments [sodium depletion; subcutaneous treatment with the combination of furosemide (FURO) + captopril (CAP) and treatment with deoxycorticosterone (DOCA)], while injections of a serotonergic agonist (DOI) reduced these intakes [11-13]. Increase of FURO+CAP-induced sodium intake is also produced by the blockade of cholecystokinin (CCK) receptors or activation of GABA_A_ and GABA_B_ receptors into the LPBN [14-16]. Therefore, in the LPBN, neurotransmitters like serotonin (5-HT) and CCK activate an inhibitory mechanism, while activation of GABAergic receptors in the LPBN is suggested to deactivate inhibitory mechanisms present in this area increasing water and 0.3 M NaCl intake.

The LPBN is also related to the inhibition of sodium intake by increased plasma osmolarity. Previous studies have shown that the blockade of LPBN inhibitory mechanisms by injections of methysergide or moxonidine combined with an increase in plasma osmolarity (intragastric load of 2 M NaCl) induced an unexpected ingestion of hypertonic NaCl [17,18], a clear paradox considering that 2 M NaCl load usually suppresses sodium appetite [19]. In addition, a previous study showed that activation of GABA_A_ receptors into the LPBN induced 0.3 M NaCl intake in cell-dehydrated rats, suggesting that the increase in plasma osmolarity subsequent to NaCl ingestion does not appear to prevent further salt intake when GABA_A_ receptors in the LPBN are activated [20].

The γ-aminobutyric acid (GABA) is the most important inhibitory neurotransmitter in the mammalian central nervous system [21,22]. GABA receptors can be classified into two structurally and pharmacologically distinct subclasses: GABA_A_ and GABA_B_[23,24]. Activation of GABA_A_ receptors leads to opening of the channel and the conductance of Cl^−^ ions and this effect may be antagonized by bicuculline [25]. GABA_B_ receptors are metabotropic, G protein-coupled receptors that mediate presynaptic and postsynaptic inhibition by reductions in calcium conductance or increases in potassium conductance [24,26]. GABA_B_ receptors are activated by baclofen and 2-hydroxysaclofen antagonizes these receptors [26,27].

A dense plexus of GABA-immunoreactive varicosities has been shown throughout the parabrachial nucleus (PBN) and Kolliker fuse (KF) complex [28]. Both GABA_A_ and GABA_B_ receptors are present in LPBN [29,30] and a previous study showed that baclofen injected into LPBN induced ingestion of 0.3 M NaCl and water in fluid replete rats [31]. In addition, injections of baclofen into the LPBN increased 0.3 M NaCl and water intake by rats injected subcutaneously with the diuretic furosemide combined with the angiotensin converting enzyme inhibitor captopril [16].

Considering the possibility that baclofen deactivates the inhibitory mechanisms and increases 0.3 M NaCl intake in cell-dehydrated rats like that produced by GABA_A_ receptor activation in the LPBN, in the present study, we investigated the effects of bilateral injections of baclofen alone or combined with the GABA_B_ receptor antagonist 2-hydroxysaclofen into the LPBN on 0.3 M NaCl and water intake in rats with increased plasma osmolarity and compared the responses with the effects of baclofen into the LPBN on sodium intake in fluid replete rats that received no pre-treatment. In addition, possible changes in cardiovascular responses to baclofen injected into the LPBN in cell-dehydrated rats were also investigated. Intragastric load of 2 M NaCl produces cell dehydration by increasing plasma osmolality and sodium concentration and also reduces plasma renin activity [32].

## Materials and methods

### Animals

Male Wistar rats weighing 290–310 g were used. The animals were housed in individual stainless steel cages with free access to normal sodium diet (Guabi Rat Chow, Paulinia, SP, Brazil), water and 0.3 M NaCl solution. The position of the bottles containing water and 0.3 M NaCl was rotated daily to avoid place preference. Room temperature was maintained at 23 ± 2°C and humidity at 55 ± 10% in a 12:12 light–dark cycle with light onset at 07:30 AM. The Institutional Ethics Committee on Animal Care and Use of the School of Dentistry, Araçatuba–UNESP approved the experimental protocols used in the present study (Proc. CEEA No. 057/03) and followed the recommendations of the Brazilian College of Animal Experimentation (COBEA). The procedures were in compliance with the National Institutes of Health Guide for the Care and Use of Laboratory Animals (NIH publication No. 80–23, 1996, USA). All efforts were made to minimize animal discomfort and the number of animals used.

### Cerebral cannulas

Rats were anesthetized with ketamine (80 mg/kg of body weight, Agener União, Embu-Guaçu, SP, Brazil) combined with xylazine (7 mg/kg of body weight, Agener União, Embu-Guaçu, SP, Brazil ) and placed in a Kopf stereotaxic instrument (Kopf, Tujunga, CA, USA). The skull was leveled between bregma and lambda. Bilateral stainless steel 23-gauge cannulas were implanted in direction to the LPBN using the following coordinates: 9.4 mm caudal to bregma, 2.2 mm lateral to the midline, and 3.9 mm below the dura mater. The tips of the cannulas were put into place at a point 2 mm above each LPBN. The cannulas were fixed to the cranium using dental acrylic resin and jeweler screws. A 30-gauge metal obturator filled the cannulas between tests. The experiments began 5 days later when the animals had fully recovered from the surgery.

### Injections into the LPBN

Bilateral injections into the LPBN were made using 5-μl Hamilton syringe connected by polyethylene tubing (PE-10) to 30-gauge injection cannula. At the time of testing, the obturators were removed and the injection cannula (2 mm longer than the guide cannula) was carefully inserted into the guide cannula, and manual injection was initiated 15 s later. For bilateral injections, the first injection was initially performed in one side, the needle was withdrawn and repositioned on the contralateral side, and then the second injection was made. Therefore, injections were made ~1 min apart. The injection volume into the LPBN was 0.2 μl in each site. The obturators were replaced after the injections, and the rats were put back into their cages.

### Drugs

The drugs injected into the LPBN were (±)-baclofen and 2-hydroxysaclofen purchased from Sigma-Aldrich (Saint Louis, MO, USA) and were dissolved in 0.15 M NaCl. The doses of baclofen and 2-hydroxysaclofen were chosen based on a previous study [31]. Doses of baclofen produce a long-lasting action (at least for three hours) when injected into the LPBN. The dose of 2-hydroxysaclofen used was an effective dose that blocks GABA_B_ receptors into LPBN as showed previously [31].

### Water and 0.3 M NaCl intake

The rats were tested in their home cages. In addition to water and food pellets, rats had access to 0.3 M NaCl for at least 5 days before the experiments began. The effects of baclofen were tested under three different experimental conditions: 1) In the same group of rats (n=9), we evaluated if baclofen affect water and 0.3 M NaCl intake in rats that received no pre-treatment (fluid replete rats) and after intragastric load of 2 M NaCl (cell-dehydrated condition). First, fluid replete rats received injections of baclofen (0.5 nmol/0.2 μl) or saline into the LPBN and 15 min later rats were given water and 0.3 M NaCl in graduated (0.1 ml divisions) glass burettes. Cumulative water and 0.3 M NaCl intake was measured at each 30 min time interval in the next 210 min after the access to water and sodium solution. The same group of animals, received an intragastric load of 2 M NaCl, to induce cellular dehydration. Forty-five minutes later, half of the group received injections of baclofen (0.5 nmol/0.2 μl), and the other half received saline into the LPBN and 15 min later rats had free access to both water and 0.3 M NaCl (two-bottle test). Cumulative water and 0.3 M NaCl intake was recorded at each 30 min time interval in the next 210 min after the access to water and sodium solution. The same procedure was repeated in a second experimental session performed 3 days later in a counterbalanced design. All tests began between 13:00 p.m. and 16:00 p.m.

2) In another group of cell-dehydrated rats (n=11), the effects of two different doses of baclofen (0.5 and 1.0 nmol/0.2 μl) injected into the LPBN on water and 0.3 M NaCl intake were tested. Rats received an intragastric load of 2 M NaCl. Forty-five minutes later, half of the group received baclofen (0.5 or 1.0 nmol/0.2 μl), and the other half received saline injections into the LPBN. The same procedure was repeated in a second experimental session performed 3 days later in a counterbalanced design. Fifteen minutes after LPBN injections, the animals had free access to both water and 0.3 M NaCl. Cumulative water and 0.3 M NaCl intake was recorded at each 30, 60, 90, 120, 180, and 210 min after the access to water and sodium solution. All tests began between 13:00 p.m. and 16:00 p.m.

3) To test a possible participation of GABA_B_ receptors in the effects of baclofen, another group of rats (n=6) received an intragastric load of 2 M NaCl 45 min before bilateral injections of baclofen (0.5 nmol/0.2 μl) or saline into the LPBN, and cumulative water and 0.3 M NaCl intake was measured at each 30 min in the next 210 min, starting 15 min after the injections of baclofen or saline into the LPBN. This group of rats also received bilateral injections of 2-hydroxysaclofen (5 nmol/0.2 μl) or saline into the LPBN, 15 min before baclofen or saline injections. This group of rats was submitted to four tests. In each test the group was divided in two subgroups that received different combinations of treatments into the LPBN. The sequence of the combinations of treatments into the LPBN in each rat in different tests was randomized, and at the end of the tests each rat received all the combinations of treatments into the LPBN. All tests began between 13:00 p.m. and 16:00 p.m.

### Arterial pressure and heart rate recordings

Mean arterial pressure (MAP) and heart rate (HR) were recorded in unanesthetized rats. One day before recording, the rats were anesthetized with ketamine (80 mg/kg of body weight) + xylazine (7 mg/kg of body weight), a polyethylene tubing (PE 10 connected to a PE 50) was inserted into the abdominal aorta through the femoral artery for arterial pressure recording. The cannula was guided subcutaneously and exteriorized at the back of the rat. On the next day, the cannula was connected to a P23 Db pressure transducer (Statham Gould) coupled to a pre-amplifier (model ETH-200 Bridge Bio Amplifier, CB Sciences) connected to a Powerlab computer recording system (Powerlab 8SP, ADInstruments) to record MAP and HR. The animals received an intragastric 2 M NaCl load and forty-five minutes after this, one group of rats was tested for the effects of baclofen (1.0 nmol/0.2 μl) and another group for the effects of saline injected into the LPBN on MAP and HR. MAP and HR were recorded for the next 180 minutes after injections into LPBN and the maximum changes were analyzed. During MAP and HR recordings, the rats did not have access to water, 0.3 M NaCl and food.

### Histology

On completion of the experiments, the animals received bilateral injections of 0.2 μl of 2% Evans blue dye into the LPBN. Then, they were deeply anesthetized with sodium thiopental (80 mg/kg of body weight) and perfused transcardially with saline followed by 10% formalin. The brains were removed, fixed in 10% formalin, frozen, cut in 60 μm serial coronal sections, stained with Giemsa, and analyzed by light microscopy to confirm the injection sites into the LPBN.

### Statistical analysis

The results are reported as means ± S.E.M. Water and 0.3 M NaCl intake were analyzed by two-way analysis of variance (ANOVA) with repeated measures for both factors (treatments and times), followed by Newman-Keuls post hoc test. Significance was set at *P* < 0.05. The software used to analyze the data was SigmaStat for Windows, version 2.03 from SPSS Inc.

## Results

### Histological analysis

**Figure 1:**
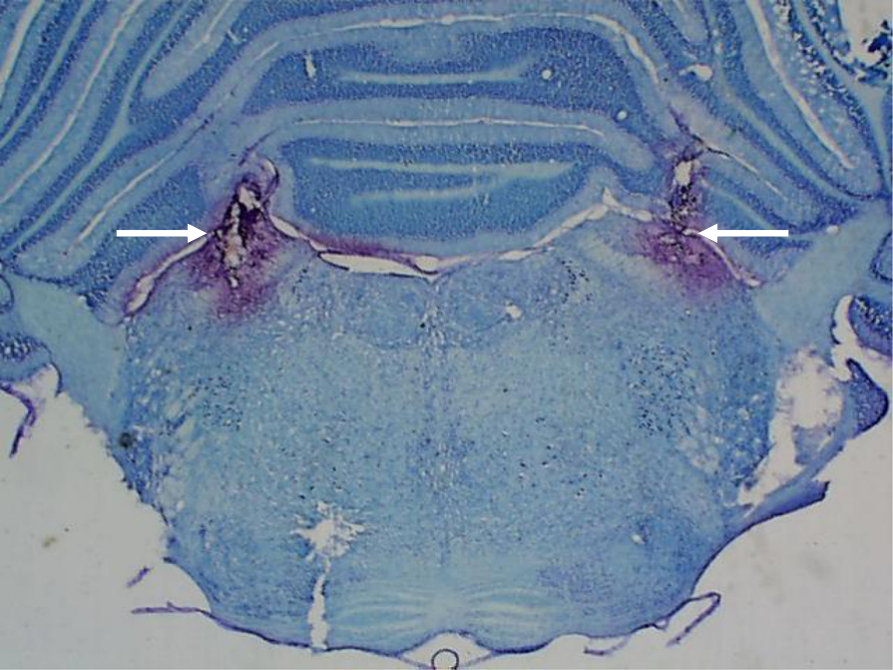
Photomicrograph showing the sites of injections into the LPBN (arrows).

Similar to previous reports [16,20,31], the LPBN injection sites were centered in the central lateral and dorsolateral portions of the LPBN. Figure 1 shows the typical LPBN injection sites. Injections reaching the ventral lateral and external lateral portions, as well as the Kolliker-Fuse nucleus, were observed in some rats and the results from these rats were included in the analysis. In some rats, injections also spread to the brachium (superior cerebellar peduncle), or slightly ventral to this structure, reaching the dorsal portions of the medial parabrachial nucleus (MPBN) uni- or bilaterally. There was no difference in the effects whether injections were restricted to the LPBN or also spread to brachium and dorsal portions of MPBN.

### Effects of bilateral injections of baclofen into the LPBN on water and 0.3 M NaCl intake in fluid replete and cell-dehydrated rats

**Figure 2:**
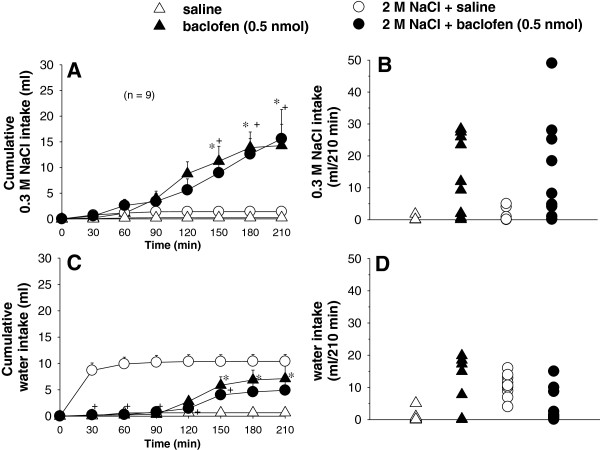
**(A,C) Cumulative and (B,D) individual 0.3 M NaCl and water intake in fluid replete rats and in rats treated with i.g. 2 M NaCl load combined with injections of baclofen or saline into the LPBN.** (**A** and **C**) Values are means ± S.E.M. n = Number of rats. * different from saline; + different from 2 M NaCl + saline. Fewer symbols appear in **B** and **D** because of overlapping data.

Bilateral injection of baclofen (0.5 nmol/0.2 μl, n=9) into the LPBN in fluid replete rats induced 0.3 M NaCl and water intake from 150 to 210 min of test (Figure 2A, C). In rats treated with an intragastric load of 2 M NaCl, baclofen injected into the LPBN increased 0.3 M NaCl intake from 150 to 210 min of test (Figure 2A, B) and reduced water intake from 30 to 150 min of test (Figure 2C, D). ANOVA showed significant interaction between treatments and time for 0.3 M NaCl intake [*F*(18, 144) = 4.3; *P* < 0.001, Figure 2A, B] and water intake [*F*(18, 144) = 3.3; *P* < 0.001, Figure 2C, D) in fluid replete rats and cell-dehydrated rats that received baclofen into the LPBN.

A hypotonic mixture of water and 0.3 M NaCl was ingested throughout the experimental session when fluid replete rats received saline in the LPBN. On an average, the rats ingested 0.2 ± 0.2 ml of 0.3 M NaCl versus 0.6 ± 0.5 ml of water in 210 min, which yielded a solution of approximately 0.09 M. However, fluid replete rats that received baclofen into the LPBN ingested 14.3 ± 4.1 ml of 0.3 M NaCl and 7.1 ± 2.9 ml of water in 210 min, which results in a hypertonic solution (0.2 M) (Figure 2A, C).

**Figure 3:**
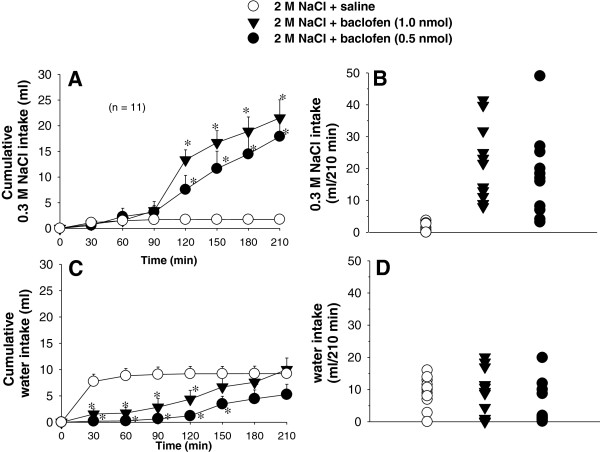
**(A,C) Cumulative and (B, D) individual 0.3 M NaCl and water intake in rats treated with i.g. 2 M NaCl load combined with injections of baclofen or saline into the LPBN.** (**A** and **C**) Values are means ± S.E.M. n = Number of rats. * different from 2 M NaCl + saline. Fewer symbols appear in **B** and **D** because of overlapping data.

After intragastric load of 2 M NaCl, bilateral LPBN injections of baclofen (0.5 and 1.0 nmol/0.2 μl) into the LPBN induced ingestion of 0.3 M NaCl from 120 to 210 min of test (Figure 3A, B), and an inhibition of water intake from 30 to 120 min of test (Figure 3C, D). There were no significant differences in 0.3 M NaCl intake [*F*(1,10) = 1.1; P > 0.05, Figure 3A, B] and water intake [*F*(1,10) = 2.8; P > 0.05, Figure 3C, D], in cell-dehydrated rats treated with different doses of baclofen (0.5 and 1.0 nmol/0.2 μl) into the LPBN.

Cell-dehydrated rats (n=11) that received saline in the LPBN ingested 1.7 ± 0.8 ml of 0.3 M NaCl and 9.2 ± 1.4 ml of water in 210 min. Considering the total intake, the result is a hypotonic solution (nearly 0.05 M). Cell-dehydrated rats treated with baclofen (0.5 nmol/0.2 μl) into the LPBN ingested 17.9 ± 4.2 ml of 0.3 M NaCl and 5.2 ± 1.9 ml of water in 210 min, which results in a hypertonic solution (nearly 0.23 M). Cell-dehydrated rats treated with baclofen (1.0 nmol/0.2 μl) into the LPBN also ingested a hypertonic solution (0.21 M).

### Effects of the combination of 2-hydroxysaclofen and baclofen into the LPBN on water and 0.3 M NaCl intake in cell-dehydrated rats

**Figure 4:**
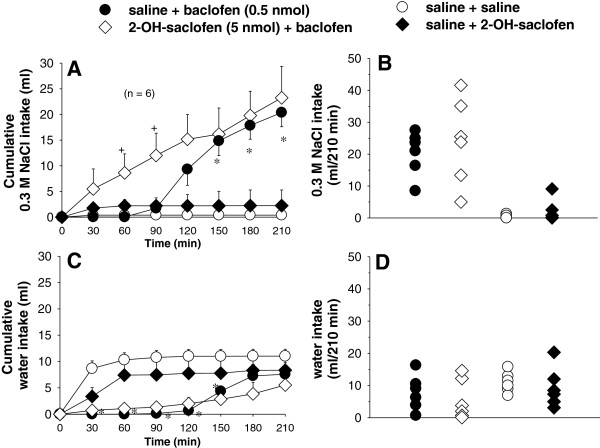
**(A,C) Cumulative and (B,D) individual 0.3 M NaCl and water intake in rats treated with i.g. 2 M NaCl load followed by injections of 2-hydroxysaclofen or saline combined with baclofen or saline into the LPBN.** (**A** and **C**) Values are means ± S.E.M. n = Number of rats. * different from saline + saline; + different from saline + baclofen. Fewer symbols appear in **B** and **D** because of overlapping data.

ANOVA showed significant differences among treatments for 0.3 M NaCl intake by cell-dehydrated rats that received saline or 2-hydroxysaclofen combined with saline or baclofen into the LPBN [F(3, 15) = 9.5; P < 0.05, Figure 4A, B]. There was significant interaction between treatments and times for water intake [F(18, 90) = 2.2; P < 0.05, Figure 4C, D].

Bilateral injections of baclofen after pretreatment with saline into the LPBN induced 0.3 M NaCl intake from 150 to 210 min of the test with P values ranging from P < 0.005 at 150 min to P < 0.001 at 180 and 210 min (Figure 4A). Baclofen induced an early inhibitory effect on water intake in the first 150 min of the test with P values ranging from P < 0.05 at 30 min to P < 0.001 at 60 to 150 min (Figure 4C, D). Previous injections of 2-hydroxysaclofen (5 nmol/0.2 μl) into the LPBN increased 0.3 M NaCl intake induced by baclofen in the first 90 minutes of test (8.6 ± 3.6; 12.0 ± 4.3 vs, baclofen: 0.01 ± 0.01; 1.7 ± 0.7 ml; respectively for 60 and 90 min; P < 0.005, Figure 4A), and did not modify 0.3 M NaCl intake induced by baclofen injected in the same area from 120 to 210 min of test (P > 0.1).

Water intake after 2-hydroxysaclofen + baclofen into the LPBN did not differ from saline + baclofen into the LPBN (P > 0.5 for all times tested, Figure 4C, D). After saline + 2-hydroxysaclofen injection into the LPBN, water or 0.3 M NaCl intake did not differ from control test with saline injections into the LPBN (P > 0.1 at any of the times studied).

### Effects of bilateral injections of baclofen into the LPBN on arterial pressure and HR in cell-dehydrated rats

Bilateral injections of baclofen (1 nmol/0.2 μl) into the LPBN in normotensive rats (basal MAP: 120 ± 4.2 mmHg and basal HR: 363 ± 6.6 beats per min, bpm), previously treated with intragastric load of 2 M NaCl, did not affect changes in MAP (3.7 ± 3.8 vs. saline: 4.9 ± 1.5 mmHg, n=7), [F (1, 6) = 0.1; P > 0.05] neither changes in HR (1.5 ± 12.7 vs. saline: 6.7 ± 13.7 bpm), [F (1, 6) = 0.5; P > 0.05].

### Specificity of injections into the LPBN to produce the effects on water and 0.3 M NaCl intake in cell-dehydrated rats

The specificity of the LPBN as the site where baclofen produced the effects on water and 0.3 M NaCl intake in cell-dehydrated rats was confirmed by results from rats in which the injections did not reach the LPBN (misplaced injections). Bilateral injections of baclofen (0.5 nmol), 2-hydroxysaclofen (5 nmol) or 2-hydroxysaclofen combined with baclofen outside the LPBN produced no effect on 0.3 M NaCl or on water intake (Table 1). ANOVA showed no significant difference among treatments for 0.3 M NaCl intake [*F*(3, 15) = 0.54; *P* > 0.05] or water intake [*F*(3, 15) = 1.69; *P* > 0.05] by cell-dehydrated rats that received injections in sites outside the LPBN (Table 1). Injections outside the LPBN reached different sites; the majority were dorsal or caudal to the LPBN, although a few were ventral or rostral to the LPBN.

**Table 1:** Ingestion of water and 0.3 M NaCl by cell-dehydrated rats treated with saline or 2-hydroxysaclofen combined with saline or baclofen in sites outside the LPBN (misplaced injections)

**Treatments**	***n***	**0.3 M NaCl intake**	**Water intake**
		**(ml/210 min)**	**(ml/210 min)**
Saline + saline	6	2.8±1.3	8.9±1.4
Saline + baclofen (0.5 nmol)	6	3.1±1.7	6.5±2.8
2-hydroxysaclofen (5 nmol) +			
baclofen	6	3.9±1.8	7.9±2.8
2-hydroxysaclofen (5 nmol) +			
saline	6	3.6±2.3	8.3±2.2

Values are means ± S.E.M.; n, number of rats.

## Discussion

In the present study, we showed that bilateral injections of baclofen into the LPBN induced both 0.3 M NaCl and water intake in rats that received no pre-treatment. Bilateral injections of baclofen into the LPBN induced ingestion of 0.3 M NaCl (from 120 to 210 min of test) and inhibited the large increases in water intake in hyperosmotic cell-dehydrated rats. At the end of 210 min of test, fluid replete rats and cell-dehydrated rats ingested a similar amount of 0.3 M NaCl after administration of baclofen into the LPBN despite of the hyperosmolality, hypernatremia, reduction of plasma renin activity and normovolemia present in cell-dehydrated rats [32]. Results from rats with misplaced injections confirmed that baclofen produces effects on water and 0.3 M NaCl intake if injected specifically in the LPBN and not into the surrounding areas.

In a recent study [20], the authors also showed that the blockade of LPBN inhibitory mechanisms by injections of muscimol in this area also induced 0.3 M NaCl intake in fluid replete rats and in rats treated with intragastric 2 M NaCl load, suggesting that the increase in plasma osmolarity subsequent to NaCl ingestion does not appear to prevent further salt intake when GABA_A_ receptors in the LPBN are activated.

Therefore, the present results suggest that baclofen injected into the LPBN induces ingestion of 0.3 M NaCl irrespective of the initial physiological state of the rats. A possible conclusion from these results is that it is not necessary to activate the excitatory mechanisms by baclofen into the LPBN for NaCl intake, which differs from the mechanisms that have been proposed to explain increased sodium intake after the blockade of LPBN inhibitory mechanisms [11-13].

Peripheral administration of baclofen reduce water deprivation-induced water intake and reduce water intake in rats pretreated with a hypertonic solution of NaCl [33,34]. The results obtained in this study on the effects of baclofen injected into the LPBN on water intake in dehydrated rats are therefore similar to those previously reported [33]. Ebenezer et al. [34] also demonstrated that systemic administration of baclofen had no effects on water intake in fluid replete rats. Interestingly, in the present study was demonstrated that bilateral injection of baclofen into the LPBN induced water and 0.3 M NaCl intake (150 – 210 min of test) in a two-bottle test in fluid replete rats. Recently [31], we showed that injections of baclofen into the LPBN induced no water intake if only water was available. As indicated by the present results, ingestion of water usually increased after injections of baclofen into the LPBN when fluid replete rats simultaneously ingested 0.3 M NaCl. In spite of the simultaneous ingestion of water, this intake is not enough to compensate for the increased osmolarity produced by the ingestion of hypertonic NaCl. The fluid ingested (water and 0.3 M NaCl), after injections of baclofen into the LPBN, was hypertonic during most of the test. Therefore, it seems that the increase in plasma osmolarity due to the ingestion of hypertonic NaCl may be reinforcing the effect of baclofen on sodium intake instead of inducing water intake.

We suggest that the effect of baclofen injected into the LPBN on water and sodium intake was due to a direct effect of baclofen on the LPBN. Callera et al. [15] demonstrated that bicuculline injected into the LPBN 60 min after muscimol strongly reduced the effects of muscimol on 0.3 M NaCl and water intake, suggesting that muscimol and probably baclofen is acting at this moment releasing water and sodium intake. On the contrary, baclofen into the LPBN reduced water intake in cell-dehydrated rats (30–150 min of test). Therefore, the effects on 0.3 M NaCl and water intake were due to baclofen facilitating the actions on GABA receptors.

The natriorexigenic effect of baclofen might be a secondary effect of baclofen injected into the LPBN, and reductions in blood pressure or increases in renal excretion would be one possibility. However, baclofen injected into the LPBN produces non-significant effects on blood pressure and renal excretion in fluid replete rats and cell-dehydrated rats [31], present data], suggesting that the natriorexigenic response to baclofen injected into the LPBN is not secondary to cardiovascular responses or increased renal excretion. It seems that injections of baclofen, similar to the injections of muscimol into the LPBN [20], completely block the action of all the inhibitory mechanisms present in the LPBN, which combined with any residual facilitatory signal still present as the one produced by normal levels of ANG II may facilitate water and especially NaCl intake.

Recently [16], we showed that pretreatment of the LPBN with bilateral injections of the nonpeptide AT_1_ receptor antagonist losartan reduced 0.3 M NaCl and water intake caused by baclofen injected into the same site in fluid replete rats, as well as the increase in water and 0.3 M NaCl produced by baclofen injected bilaterally into the LPBN in FURO+CAP-treated rats, suggesting that angiotensinergic mechanisms in the LPBN are essential for the dipsogenic and natriorexigenic responses induced by the blockade of LPBN neurons with baclofen in fluid replete rats or FURO+CAP-treated rats. It is possible that ANG II acting on AT_1_ receptors in hyperosmotic cell-dehydrated rats is not sufficient to facilitate water intake produced by baclofen in the LPBN. The ingestion of 0.3 M NaCl after baclofen injections into the LPBN takes at least 2 h to start, which is a time enough for changes in the levels of ANG II that acting in the LPBN may intensify the effects of baclofen on LPBN neurons, a step necessary for the release of sodium intake. More studies are necessary to investigate the effects of previous injection of AT_1_ receptor antagonist on water and sodium intake induced by baclofen injected into the LPBN in hyperosmotic cell-dehydrated rats.

The present study also showed that blockade of the GABA_B_ receptors with 2-hydroxysaclofen alone into the LPBN did not affect sodium intake in cell-dehydrated rats, suggesting that GABAergic mechanisms in the LPBN do not tonically inhibit or facilitate 0.3 M NaCl intake [15,20,31]. In addition, previous blockade of the GABA_B_ receptors with 2-hydroxysaclofen injected into the LPBN produced no effect on late 0.3 M NaCl intake and inhibition of water intake induced by baclofen injected in the same area. Although still not completely clear, the blockade of GABA_B_ receptor potentiated the natriorexigenic effect of baclofen in the first 90 min of the test. An involvement of GABA_A_ and GABA_B_ receptors in the natriorexigenic effect of baclofen has previously been reported [31]. The natriorexigenic effect of baclofen injected into the LPBN in fluid replete rats was reduced by the pre-treatment with the GABA_B_ receptor antagonist 2-hydroxysaclofen or by the GABA_A_ receptor antagonist bicuculline injected into the same area. Perhaps, the activation of GABA_B_ receptors by baclofen injected into the LPBN together with a baseline activation of GABA_A_ receptors by endogenous GABA produces a sufficient inhibition of LPBN mechanisms to release sodium intake. Or instead of endogenous GABA, a nonspecific activation of a limited number of GABA_A_ receptors by baclofen together with the activation of GABA_B_ receptors in the LPBN might inhibit the LPBN mechanisms releasing NaCl intake. In both cases, although only the activation of GABA_B_ receptors produces no sodium intake, the activation of GABA_B_ receptors in the LPBN may facilitate the effects of GABA_A_ receptor activation on sodium intake. An involvement of GABA_A_ receptors on baclofen effects was already reported. The inhibition of food intake by intraperitoneal administration of baclofen was abolished by previous treatment with bicuculline, suggesting a possible involvement of GABA_A_ receptors in the effects of baclofen [35].

Thus, the present results show that the blockade of LPBN mechanisms with baclofen induces hypertonic NaCl intake and reduces water intake in cell-dehydrated rats and induces hypertonic NaCl intake and reduces urinary sodium excretion and diuresis in fluid replete rats [31], all responses consistent with an action of LPBN mechanisms against body fluid volume expansion as previously proposed for the serotonergic mechanism in the LPBN [36]. Thus, further studies in rats submitted to intracellular dehydration are needed to test the effects of GABA receptors of the LPBN on behavioral and renal functions that induce to volume expansion.

## Conclusion

In conclusion, the results of our experiments show that fluid replete rats or hyperosmotic cell-dehydrated rats ingest similar amounts of 0.3 M NaCl after bilateral injections of baclofen into the LPBN. This observation means that, after the blockade of LPBN inhibitory mechanisms with baclofen, hypertonic NaCl intake occurs independent of whether plasma renin levels are normal or reduced and independent of changes in blood pressure.

## Abbreviations

(ANG II): Angiotensin II; (AP): Area postrema; (bpm): Beats per minute; (b. wt.): Body weight; (CAP): Captopril; (CCK): Cholecystokinin; (CRF): Corticotrophin release factor; (DOCA): Deoxycorticosterone acetate; (DOI): 2,5-dimetoxy-4-iodoamphetamine hydrobromide; (FURO): Furosemide; (GABA): Gamma-aminobutyric acid; (HR): Heart rate; (i.g.): Intragastric; (LPBN): Lateral parabrachial nucleus; (MAP): Mean arterial pressure; (NaCl): Sodium chloride; (OVLT): Organum vasculosum of the lamina terminalis; (SCP): Superior cerebellar peduncle

## Competing interests

The authors declare that they have no competing interests.

## Authors’ contributions

All the authors have made a substantial contribution to the conception and design of the study (EHK, LBO, JVM and JCC), the acquisition, analysis and interpretation of the data (EHK and JCC) and the drafting and revision of the article (LBO, JVM, and JCC). All the authors read and approved the final manuscript.
